# No association between 25-hydroxyvitamin D levels and prediabetes in Brazilian patients. A cross-sectional study: Ausência de associação entre níveis de 25-hidroxivitamina D e pré-diabetes em pacientes brasileiros. Um estudo transversal

**DOI:** 10.1590/1516-3180.2013.7180005

**Published:** 2014-11-28

**Authors:** Guilherme de Vieira Giorelli, Lívia Nascimento de Matos, Amir Saado, Vera Lúcia Soibelman, Cristiane Bitencourt Dias

**Affiliations:** I MD. Master’s Student. Hospital do Servidor Público Estadual de São Paulo (HSPE), São Paulo, Brazil.; II MD, MSc. Attending Physician, Department of Cardiology, Universidade Federal de São Paulo (Unifesp), São Paulo, Brazil.; III MD. Head of the Clinical Medicine Service, Hospital do Servidor Público Estadual de São Paulo (HSPE), São Paulo, Brazil.; IV MD, MSc, PhD. Attending Physician, Department of Clinical Medicine, Hospital do Servidor Público Estadual de São Paulo (HSPE), São Paulo, Brazil.

**Keywords:** Vitamin D, Glucose intolerance, Prediabetic state, Hyperglycemia, Diabetes mellitus

## Abstract

**CONTEXT AND OBJECTIVE:**

Several studies have evaluated the role of low 25-hydroxyvitamin D (25OHD3) in the pathogenesis of type 2 diabetes (T2DM) and have presented controversial results. The metabolic processes that culminate in T2DM begin under prediabetic conditions. Our aim was to analyze the association between 25OHD3 and glucose metabolism in individuals who were free from but at elevated risk of diabetes.

**DESIGN AND SETTING:**

Cross-sectional study at a tertiary hospital.

**METHODS:**

Anthropometric and laboratory profiles were determined in patients with one or more of the following risk factors: hypertension; body mass index (BMI) ≥ 25 kg/m^2^; waist circumference > 80 cm for women and > 94 cm for men; first-degree relatives with diabetes; women with large-for-gestational-age newborns or with gestational T2DM; HDL-cholesterol (high density lipoprotein) < 35 mg/dl; and triglycerides > 250 mg/dl. The patients were divided into two groups: one with prediabetes (abnormal fasting plasma glucose or oral glucose tolerance test) and the other with normal glucose (euglycemic).

**RESULTS:**

There was no statistically significant difference between the prediabetic group (n = 38) and euglycemic group (n = 15) regarding age (66.4 ± 10.6 versus 62.6 ± 9.1 years), gender (52.6 versus 73.3% female) and BMI (30.1 ± 4.61 versus 27.9 ± 4.7 kg/m^2^). Low serum levels of 25OHD3 were found in both groups, without any statistically significant difference between them (29.1 ± 11.8 versus 26.87 ± 9.2 ng/dl).

**CONCLUSION:**

There was no association between 25OHD3 levels and the clinical or laboratorial variables analyzed.

## RESUMO

**CONTEXTO E OBJETIVO:**

Vários estudos já avaliaram o papel da 25-hidroxivitamina D (25OHD3) na patogênese do diabetes tipo 2 (DM2) e apresentaram resultados controversos. Os processos metabólicos que culminam no DM2 se iniciam no pré-diabetes. Nosso objetivo foi analisar a associação da 25OHD3 com o metabolismo glicêmico em indivíduos sem diagnóstico mas com alto risco para diabetes.

**TIPO DE ESTUDO E LOCAL:**

Estudo transversal em hospital terciário.

**MÉTODOS:**

Medidas antropométricas e laboratoriais foram determinadas em pacientes com um ou mais dos fatores de risco: hipertensão; índice de massa corpórea (IMC) ≥ 25 kg/m^2^; circunferência abdominal > 80 cm no sexo feminino e > 94 cm no sexo masculino; parentes de primeiro grau com diabetes; mulheres com filho nascido grande para idade gestacional ou com DM2 na gravidez; colesterol HDL (*high density lipoprotein*) < 35 mg/dl e triglicerídeo > 250 mg/dl. Os pacientes foram divididos em dois grupos: um com pré-diabetes (glicemia de jejum ou teste de tolerância oral à glicose alterados) e outro com glicose normal (euglicêmicos).

**RESULTADOS:**

Entre pré-diabéticos (n = 38) e euglicêmicos (n = 15) não houve diferença estatística na idade (66,4 ± 10,6 *versus* 62,6 ± 9,1 anos), gênero (52,6 *versus* 73,3% feminino) e IMC (30,1 ± 4,61 *versus* 27,9 ± 4,7 kg/m^2^). Baixos niveis séricos de 25OHD3 foram encontrados nos dois grupos, sem diferença estatística entre eles (29,1 ± 11,8 *versus* 26,87 ± 9,2 ng/dl).

**CONCLUSÃO:**

Não houve associação entre os níveis de 25OHD3 e as variáveis clínicas e laboratoriais analisadas.

## INTRODUCTION

The presence of vitamin D receptors (VDRs) has been described in pancreas β cells, adipose tissue and muscle tissue. Some studies on animals have suggested that vitamin D is involved in the synthesis and secretion of insulin, which might indicate that lower serum 25-hydroxyvitamin D (25OHD3) could have an impact on type 2 diabetes (T2DM).^1^^,^^2^

Twelve million Brazilians presented T2DM in 2011, according to official Brazilian government data, and the prevalence of low serum 25OHD3 has increased all over the world in the last few years. Recently, low serum 25OHD3 has been considered to be a worldwide public health issue due to its impact on many diseases.^3^ Despite great sunlight exposure in Brazil, low serum 25OHD3 has been reported. Saraiva et al.^4^ and Genaro et al.^5^ found unsuitable serum concentrations in 42% of the elderly in the city of São Paulo and 24% of osteoporotic women. In healthy teenagers and young adults, the prevalence was 60% and 50% respectively.^6^^,^^7^

Since prediabetes is an early stage of T2DM in which the metabolic process has already started, prevention efforts at this stage have greater impact in mitigating the development of T2DM.^8^^,^^9^ Several clinical studies have evaluated the role of low 25OHD3 in the pathogenesis of T2DM, but with controversial results.^10^^,^^11^^,^^12^^,^^13^^,^^14^^,^^15^ Recently, a study has looked into prediabetes and 25OHD3 in a representative population in the United States. This study reported that low serum 25OHD3 levels were associated with prediabetes in that population, with a strong correlation when vitamin D levels were lower than 17 ng/ml.^16^

To try to prove the association between vitamin D and glucose metabolism, Davidson et al. conducted a randomized study on vitamin D replacement in patients with prediabetes and hypovitaminosis D, and demonstrated that vitamin D replacement taken for one year did not have any benefit in relation to glucose metabolism.^17^

## OBJECTIVE

Since the findings regarding the association between vitamin D levels and glucose metabolism continue to be conflicting, and because so far no studies on prediabetes and vitamin D have been produced in any South American country, we focused on analyzing vitamin D levels in subjects who were free from diabetes but at elevated risk of T2DM and the correlation with glucose metabolism, in a tertiary-level hospital in the city of São Paulo.

## METHODS

We carried out a cross-sectional study from December 2009 to July 2010 in an outpatient clinic in the Department of Internal Medicine of a tertiary-level hospital in São Paulo, Brazil.

The subjects gave their written informed consent to participate in the study. The study was approved by the research ethics committee of Hospital do Servidor Público Estadual de São Paulo (HSPE) (protocol number 0010.338.000-08).

### Inclusion criteria

The study included individuals who were free from T2DM, but with at least one of the following conditions that have been correlated with higher risk of developing diabetes *mellitus*: hypertension; body mass index (BMI) ≥ 25 kg/m^2^; waist circumference > 80 cm for women and > 94 cm for men; first-degree relatives with diabetes; women with large-for-gestational-age newborns or with gestational diabetes *mellitus*; fasting serum HDL-cholesterol < 35 mg/dl; and triglycerides > 250 mg/dl. Since the Brazilian population is one of the most mixed in the world, ethnic groups were not considered as an inclusion criterion alone.

### Exclusion criteria

The exclusion criteria were defined as follows: prior diagnosis of diabetes *mellitus*; use of oral hypoglycemic agents or insulin; use of vitamin D or calcium supplementation; and glomerular filtration rate lower than 30 ml/min/1.73 m^2^. Changes in lifestyle such as treatment for obesity, dyslipidemia, metabolic syndrome or any pathological condition did not constitute exclusion criteria. The only lipid-lowering drugs that were used by some of the study subjects were statins.

The diagnosis of diabetes *mellitus* was defined as random plasma glucose > 200 mg/dl for symptomatic patients, and for asymptomatic patients, two results on different days with fasting plasma glucose (FPG) > 125 mg/dl or two-hour glucose ≥ 200 mg/dl after oral administration of 75 g of glucose overload (GTT). Prediabetes was defined as FPG of 100-125 mg/dl or two-hour glucose of 140-199 mg/dl after oral administration of 75 g of glucose overload.

The diagnosis of metabolic syndrome was defined in cases of occurrence of three out of the following five clinical conditions: fasting plasma glucose equal or higher than 100 mg/dl, blood pressure equal or higher than 130/85 mmHg, triglycerides equal or higher than 150 mg/dl, HDL < 40mg/dl in males and < 50 mg/dl in females, and waist circumference ≥ 102 cm (males) and ≥ 88 cm (females).

### Anthropometric measurements

All anthropometric measurements were performed by a single observer. Weight and height were assessed while the subjects were wearing light clothes; waist circumference (WC) was measured at the midpoint between the lower costal border of the last rib and the upper border of the iliac crest; and hip circumference (HC) was measured at the level of the greater trochanter.

### Laboratory analysis

Blood samples were collected by means of antecubital venous puncture, after the subjects had spent 12 hours fasting (overnight) and after a five-minute rest in a seated position, in a room with no sun exposure.

Prior to FPG and the two-hour glucose analysis, the subjects were instructed to adhere to a high-carbohydrate diet for three days; not to use any laxative on the day before the test; and not to do any physical exertion just before the test. If individuals presented diarrhea during the 48-hour period preceding the two-hour glucose test, it was rescheduled for another day. Individuals were also instructed to avoid walking and they were not allowed to smoke at any time during the test; ingestion of food of any kind was also prohibited during the test. The plasma glucose level was determined using an enzymatic method.

Plasma insulin levels were measured after the 12 hours of overnight fasting by means of an immunometric method in a two-sided solid-phase chemiluminescent assay (Immulite 2000, Siemens TM, Los Angeles, USA). Insulin resistance (IR) was evaluated by means of the formula for homeostasis model assessment (HOMA) of IR: HOMA-IR = (glucose x insulin)/22.5.

Serum uric acid, creatinine, triglyceride and cholesterol concentrations were obtained by means of standard methods. We also evaluated microalbuminuria (µg/min) using the chemiluminescence method, in samples of 24-hour urine. For the estimated glomerular filtration rate (GFR), we used the modification of diet in renal disease (MDRD) formula.

Serum 25-hydroxyvitamin D3 levels were assessed using a radioimmunoassay kit (DiaSorin, Stillwater, MN, USA) in which the intra-assay coefficient of variation was 8.6-12.5% and the inter-assay coefficient of variation was 8.2-11.0%.

### Statistical analysis

Statistical analyses were performed using the GraphPad Prism 4 software. The statistical significance level was set at P < 0.05. Continuous variables were expressed as the mean ± standard deviation or the median with interquartile range, for the variables with and without normal distribution, respectively. Categorical variables were expressed as percentages. The unpaired t test was used to analyze the difference between two groups, and the Mann-Whitney test was performed when the groups did not have normal distribution. Correlations were obtained through the Pearson or Spearman test, when appropriate.

## RESULTS

We evaluated 53 subjects with a mean age of 65.3 ± 10.3 years, of whom 31 (58.5%) were women. The mean BMI was 29.5 ± 4.7 kg/m^2^, WC 99.8 ± 12.9 cm and HC 104.3 ± 13.1 cm. The median number of risk factors for diabetes was 4 (4-5). The mean FG was 100.5 ± 11.2 mg/dl, GTT 132.8 ± 30.3 mg/dl, HDL-cholesterol 49.9 ± 12.9 mg/dl, LDL-cholesterol 122.2 ± 36.2 mg/dl, triglycerides 146.2 ± 7.6 mg/dl and uric acid 5.3 ± 2.4 mg/dl. The median serum insulin level was 6.9 (4.6-16.1) mUI/ml, median HOMA-IR 1.9 (0.9-4.0), mean serum creatinine 0.9 ± 0.2 mg/dl, mean glomerular filtration rate from MDRD 69.2 ± 16.7 ml/min/1.73 m^2^, median microalbuminuria 7.1 (3.6-10.4) µg/min and mean 25OHD3 was 28.4 ± 11.1 ng/ml.

Thirty-eight patients (71.7%) had prediabetes and 15 (28.3%) were euglycemic. Comparing the prediabetes group with the euglycemic group there were no differences in gender distribution (52.6 versus 73.3% females, respectively), age (66.4 ± 10.6 versus 62.6 ± 9.1 years, respectively), frequency of hypertension (71.5 versus 73.3%, respectively), frequency of dyslipidemia (77.7 versus 66.6%, respectively) or BMI (30.1 ± 4.6 versus 27.9 ± 4.7 kg/m^2^, respectively), as shown in Table 1.

**Table 1.**
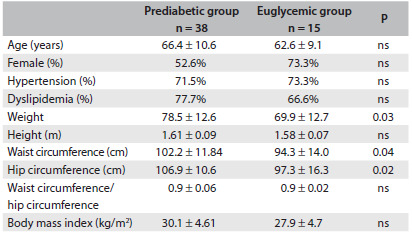
Clinical characteristics of the prediabetic and euglycemic groups

Mean ± standard deviation. ns = not significant.

Compared with the euglycemic group, the prediabetes group presented significantly higher WC (102.2 ± 11.8 versus 94.3 ± 14.0 cm, P = 0.04, respectively), HC (106.9 ± 10.6 versus 97.3 ± 16.3 cm, P = 0.02, respectively), uric acid (6.6 ±1.5 versus 5.1 ± 1.4 mg/dl, P = 0.003, respectively), serum insulin (9.6 (5.4-17.9) versus 5.7 (3.3-9.0) mUI/ml, P = 0.03, respectively), HOMA-IR (2.6 (1.2-4.7) versus 1.4 (0.7-1.7), P = 0.01, respectively) and microalbuminuria (30.6 ± 88.0 versus 5.2 ± 3.3 µg/min, P = 0.02, respectively), as shown in Table 2. Total cholesterol (225.0 ± 36.3 versus 191.8 ± 37.6 mg/dl, P = 0.005), HDL-cholesterol (55.6 ± 17.6 versus 47.6 ± 9.9 mg/dl, P = 0.04) and LDL-cholesterol (144.9 ± 31.0 versus 112.9 ± 34.4 mg/dl, P = 0.003) were higher in the euglycemic group than in the prediabetes group, respectively (Table 2). 

**Table 2.**
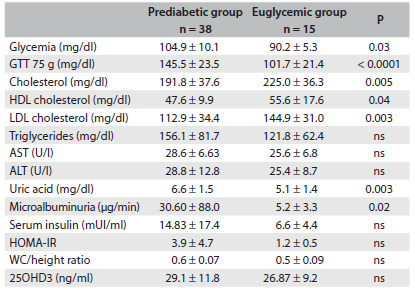
Biochemical characteristics of the prediabetic and euglycemic groups

Mean ± standard deviation. ns = not significant; GTT = glucose tolerance test; HDL = high-density lipoprotein; LDL = low-density lipoprotein; AST = aspartate aminotransferase; ALT = alanine aminotransferase; HOMA-IR = homeostasis model assessment - insulin resistance.

Low serum levels of 25-hydroxyvitamin D3 were found in both groups, with no difference between the prediabetes and euglycemic groups (29.1 ± 11.8 versus 26.8 ± 9.2 ng/ml, respectively). We did not find any correlation between serum 25OHD3 and any anthropometric parameters (weight, BMI, WC and HC). In a subanalysis on BMI groups, there was no difference in 25OHD3 levels between subjects with BMI < 30 kg/m^2^ (n = 30) and BMI ≥ 30 kg/m^2^ (n = 23): 25.9 ± 8.0 versus 30.6 ± 12.2 ng/ml, respectively.

There was no correlation between 25OHD3 levels and fasting glucose, GTT, serum insulin, HOMA-IR or other laboratory variables. In the whole group, there were 35 patients (66%) with three or more criteria for metabolic syndrome. We did not find any difference in serum 25OHD3 between groups with metabolic syndrome (n = 35) and without metabolic syndrome (n = 18): 29.2 ± 11.9 versus 26.6 ± 9.7 ng/ml, respectively.

In our sample, we had 38 patients with age ≥ 60 years old and 15 with age < 60 years old. Comparison of 25OHD3 levels between these groups did not show any difference: 29.0 ± 12.0 versus 27.1 ± 8.7 ng/ml, respectively.

## DISCUSSION

There is evidence that 3% of the human genome is managed by 1.25(OH)2D3, the active form of vitamin D.^18^ Because of the presence of vitamin D receptors in several tissues, vitamin D has been ascribed an important role in neoplasms and in the immune and cardiovascular systems.^19^^,^^20^^,^^21^ In addition, other observations indicate that hypovitaminosis D could have a relationship with the development of metabolic syndrome, including alteration of high-density lipoproteins, triglycerides and blood pressure.^22^^,^^23^

Some studies have provided evidence that vitamin D deficiency may contribute directly to or have an adjunct effect on the pathogenesis of diabetes. Although these articles showed different outcomes regarding vitamin D and diabetes, vitamin D was correlated with insulin production and secretion, and with regulation of signal transduction and decreased activity of glucose transporter-4 (GLUT 4).^1^^,^^24^^,^^25^

Scragg et al. confirmed that there was an association between low 25OHD3 and diabetes among non-Hispanic whites and Mexican Americans in the National Health and Nutrition Examination Survey (NHANES) III, and so did Liu et al. in the Framingham Study.^10^^,^^11^ The present study and others did not find any association after multivariable adjustments,^13^^,^^14^ or in subgroup analyses on women.^15^

Recently, Anoop Shankar et al.^16^ studied the relationship between serum 25-hydroxyvitamin D levels and prediabetes in an American population, using data from NHANES III. The group with prediabetes comprised 42.9% females versus 55.6% in the non-prediabetes group. These two groups also presented statistical differences in age and BMI. They found a positive association between serum 25OHD3 and prediabetes only in non-Hispanic white subjects, but not in non-Hispanic blacks, Mexican Americans and others. The strongest association was with serum vitamin D lower than 17 ng/ml.

The Brazilian population is one of the most ethnically mixed populations in the world.^26^ We conducted a study on two groups with the same gender distribution, BMI and age. Prediabetic groups had higher weight circumference, hip circumference, serum uric acid, serum insulin, HOMA-IR and microalbuminuria. These data suggest that when prediabetes is diagnosed, metabolic events that are characteristic of diabetes have already begun. Total cholesterol and LDL cholesterol were higher in the euglycemic group probably because of the lesser use of statins.

Our study did not find any association between 25OHD3 and prediabetes. This may be related to the number of individuals in our sample, given that a study that showed this relationship had a population-based sample.^16^ Moreover, despite the low levels of vitamin D in our sample, only 28.3% had values lower than 20 ng/ml (data not shown), whereas the correlation with prediabetes that has been demonstrated in the literature occurred with values lower than 17 ng/ml.^16^ However, in a recent study on 175 obese adolescents, of whom 60% were euglycemic, 25% prediabetic and 15% diabetic, Las Heras et al. did not find any difference in serum 25OHD3 between these groups, or any association with any metabolic parameters, even though the prevalence of low levels of vitamin D was higher in both groups.^27^

Another study, on 150 obese children and adolescents living in the tropics, found vitamin D levels ≥ 20 ng/ml in 88.6%, with no difference in the prevalence of prediabetes in relation to the group with levels lower than 20 ng/ml.^28^ In spite of our negative results regarding the correlation between prediabetes and vitamin D, measurement of 25OHD3 levels in patients with prediabetes is important because of the worldwide problem of vitamin D deficiency. However, more studies are necessary in order to reach a conclusion regarding the correlation between prediabetes and vitamin D deficiency.

## CONCLUSION

Our study did not find any association between 25OHD3 and prediabetes. Low serum vitamin D was found in both groups, which is consistent with the worldwide vitamin D deficiency, thus making it difficult to analyze the relationship between prediabetes and vitamin D deficiency.

Prediabetes is an early stage of diabetes. Any action that could prevent or delay the metabolic process that will lead to diabetes has great importance. Our study contributes towards the scarce literature on vitamin D and prediabetes by adding information about a South American country with an ethnically mixed population.
